# Cyclodextrins in action: Modulating *candida albicans* biofilm formation and morphology

**DOI:** 10.1016/j.btre.2025.e00912

**Published:** 2025-08-09

**Authors:** Rita Márton, Hanna Hermann, Virág Tünde Kiss, Éva Fenyvesi, Lajos Szente, Mónika Molnár

**Affiliations:** a Department of Applied Biotechnology and Food Science, Faculty of Chemical Technology and Biotechnology, Budapest University of Technology and Economics, Műegyetem rkp. 3., H-1111 Budapest, Hungary; b CycloLab Cyclodextrin R & D Laboratory Ltd. 1097 Budapest, Hungary

**Keywords:** *Candida albicans*, Biofilm formation, Cyclodextrins, Farnesol, Quorum sensing

## Abstract

The primary objective of this research was to explore the bioactive potential of cyclodextrins in attenuating biofilm formation in *C. albicans*. The concentration-dependent effects of both native cyclodextrins and specific derivatives were studied at concentrations ranging from 0.1 to 12.5 mM, to determine the mechanisms by which the extent of biofilm formation can be reduced. Besides, the efficiency of various combinations of cyclodextrins and farnesol, as an antifungal substance, were examined.

The present study revealed both stimulatory and inhibitory effects of cyclodextrins on biofilm formation, depending on the structure and concentration. The bioactive potential of randomly methylated α- and γ-CD showed significant antifungal properties, as evidenced by a reduction in the biofilm formation. In addition, randomly methylated cyclodextrins were found to significantly enhance the antifungal activity of farnesol against *C. albicans*. Consequently, their synergistic effect may provide an excellent opportunity to produce a lower dose but more effective anticandidal formulation.

## Highlights

• Randomly methylated cyclodextrins exhibited high bioactivity against *C. albicans.*
• Synergetic effect of farnesol and CD in reducing biofilm formation was demonstrated.
• Random methylated cyclodextrins increased the antifungal activity of farnesol.
• Potential applicability of RAMEA and RAMEG as anticandidal compound was explored.

## Graphical abstract

**Figure:**
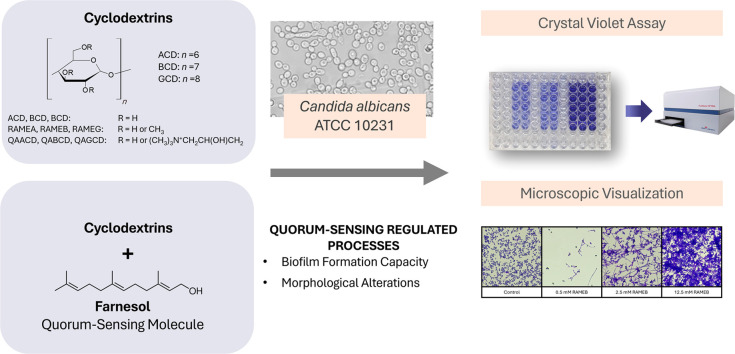

## 1 Introduction

The formation of a biofilm, a process regulated by quorum sensing (QS), is one of the most significant virulence factors exhibited by the opportunistic pathogen, *Candida albicans*. Quorum Sensing (QS) is responsible for regulating multiple processes across a variety of bacterial and fungal species [1,39]. This confers a powerful evolutionary advantage upon microbial communities, enabling rapid adaptation to changing environmental conditions. The quorum sensing of eukaryotic organisms was first discovered in the pathogenic fungus *Candida albicans* [10], which is particularly important in human medicine.

The polymorph *C. albicans* can transition from a yeast state to pseudohyphal and hyphal forms, and this behavior is regulated by a quorum sensing molecule (QSM) known as farnesol [10]. By preventing this transition, farnesol can affect the architecture and stability of biofilms [18,24,25].

*Candida albicans 10231* is the only strain reported to secrete farnesoic acid instead of farnesol [19,21,26]. Despite this, it is frequently used in research studies investigating biofilm formation and the efficacy of antifungal agents such as farnesol, as well as in quality control, thanks to its certified reference status [3,[7], [8], [9],^31^].

Moreover, other signaling molecules present in *Candida albicans*, such as tyrosol, phenylalanine, and tryptophan, also contribute to regulating important processes, including growth, morphogenesis, biofilm formation, and cell viability [15,41].

The ability of *Candida albicans* to form biofilms on various surfaces significantly enhances their chances of survival. This is primarily due to the "protected structure" of the biofilm, which is composed of polysaccharides and other materials, providing a defensive barrier. Cells within this structure exhibit substantially greater resistance compared to their counterparts in the planktonic phase, making them less vulnerable to environmental challenges, immune attacks, and antimicrobial agents [40]. As a result, infections associated with *Candida* biofilms present a considerable challenge to effective treatment and eradication using conventional antifungal therapies [20,23].

Cyclodextrins (CDs) are cyclic oligosaccharides with a unique molecular structure characterized by a hydrophobic cavity and a hydrophilic outer surface, enabling the formation of inclusion complexes with various compounds. They are widely utilized in drug and antifungal formulations to enhance the solubility, stability, and bioavailability of poorly water-soluble antifungals [11]. By forming inclusion complexes with these drugs, cyclodextrins improve their delivery, reduce toxicity, and enable more effective treatments in oral, topical, and intravenous applications [17]. A promising advancement is the development of CD-based nanocarriers, which enhance antifungal activity while maintaining favorable toxicological profiles [36]. However, their potential to exhibit intrinsic biological activity, independent of their role as carriers, has remained largely unexplored.

This gap in research is particularly significant given the ability of cyclodextrins to interact with biological membranes, potentially altering cell permeability, nutrient uptake, or signaling pathways. For example, some studies suggest that CDs may disrupt lipid membranes or influence microbial growth through mechanisms unrelated to drug delivery [13,35].

Exploring the bioactivity of CDs could reveal new mechanisms of action, provide insights into their effects on fungal physiology, and potentially identify novel therapeutic applications. Additionally, these findings could help refine the use of CDs in antifungal formulations by accounting for any unintended biological effects.

The primary objective of the present study was to evaluate the impact of cyclodextrins on *Candida albicans* yeast test system and to elucidate the underlying mechanisms. A particular focus of our studies was to assess the effects on fungal biofilm formation, which is considered to be one of the most significant virulence factors for opportunistic bacterial and fungal species. However, it is important to highlight that biofilm formation can also be beneficial in some biotechnological processes. Therefore, a key objective of our study was to assess whether CDs can influence, inhibit or stimulate this process. In bacterial systems, we have previously demonstrated the ability of CDs to influence bacterial cooperation [16]. However, there is a paucity of information on whether these environmentally friendly cyclic oligosaccharides can influence fungal communication and physiological processes.

It has been reported in several publications that farnesol plays an important role as a quorum-sensing molecule in the physiological regulatory mechanisms of *Candida* species [28,41]. In addition, it has been shown that farnesol can have significant antifungal effects on various yeasts. These effects include the inhibition of fungal hyphal growth, the induction of fungal apoptosis, the influence of ergosterol synthesis, the inhibition of activity of planktonic cells and biofilm formation [4,5,12,42,43].

In the present study, farnesol is considered an antifungal molecule of particular interest, due to the ability of cyclodextrins to complex with it [6,29,33,34]. This may provide an additional opportunity to influence biofilm formation in *Candida* species. In the context of fungi, cyclodextrins are frequently employed as excipients in antifungal formulations, with the aim of enhancing the solubility, stability, and other physicochemical properties of the active substances [13,36]. However, recent research suggests that cyclodextrins may also function as bioactive compounds, independently influencing fungal physiological processes [14].

This study investigates two quorum sensing (QS)-regulated processes of *Candida albicans*, namely biofilm formation and morphological transitions, in the presence of different cyclodextrins. The study also aims to elucidate how the combined application of farnesol and cyclodextrins can be exploited as a potential strategy to combat *Candida albicans* infection.

## 2 Materials and methods

### 2.1 Test organism and culture conditions

*Candida albicans* ATCC 10231 was maintained on YPD (10 g yeast extract l^−1^; 20 g bacteriological peptone l^−1^; 20 g glucose l^−1^) as agar slants culture. For each experiment, 30 ml of liquid YPD growth medium was inoculated with a loop of yeast colony to establish a cell culture at 30°C for 16 h (overnight). *Candida albicans* usually grows in the budding–yeast phase in these circumstances. Cells were collected from overnight liquid cultures and washed twice via centrifugation at 4000 x g in sterile phosphate-buffered saline (PBS). Subsequently, the cells were resuspended in RPMI-1640 medium (Capricorn Scientific), and their concentration was adjusted spectrophotometrically to achieve a final absorbance of 0.2 at 600 nm.

### 2.2 Characteristics of the tested molecules

We investigated the time- and concentration-dependent effects of the cyclodextrins and their derivatives, summarized in Table 1. The table includes the abbreviations of the studied cyclodextrins (with substitution degrees indicated in parentheses) and their average molecular formulae.

**Table 1:** Abbreviations (with the degree of substitution given in parenthesis) and average molecular formula of tested cyclodextrins.

Name	Abbreviation	Average molecular formula
Native α-CD	ACD	C_36_H_60_O_30_
Native β-CD	BCD	C_42_H_70_O_35_
Native γ-CD	GCD	C_48_H_80_O_40_
Randomly methylated α-CD	RAMEA (11)	C_36_H_60-n_O_30_ · (CH_3_)_n_
Randomly methylated β-CD	RAMEB (12)	C_42_H_70-n_O_35_ · (CH_3_)_n_
Randomly methylated γ-CD	RAMEG (10-13)	C_48_H_80-n_O_40_ · (CH_3_)_n_
Trimethyl-aminopropyl α-CD	QAACD (2.5-4)	C_36_H_60-n_O_30_ · (C_6_H_15_ONCl)_n_
Trimethyl-aminopropyl β-CD	QABCD (3-4)	C_42_H_70-n_O_35_ · (C_6_H_15_ONCl)_n_
Trimethyl-aminopropyl γ-CD	QAGCD (2.5-5)	C_48_H_80-n_O_40_ · (C_6_H_15_ONCl)_n_

For the experiments, the cyclodextrins (Cyclolab Ltd. Budapest, Hungary) were suspended in distilled water, sterilized by autoclaving, and then diluted to prepare a series of sterile cyclodextrin stock solutions (or suspensions). In experiments involving only cyclodextrins, the effects were evaluated at concentrations ranging from 0.5 to 12.5 mM, except for BCD, where solubility limitations restricted the concentration range to 0.1–2.5 mM. Sterile distilled water served as the negative control in these measurements.

For measurements involving farnesol, cyclodextrins were used at a final concentration of 12.5 mM, except for BCD, which was tested at 2.5 mM. Farnesol (Sigma-Aldrich Inc. Budapest, Hungary) was dissolved in methanol and used at a 1250 µM- 62.5 µM concentration range. Since farnesol was dissolved in methanol for these experiments, methanol-matched controls were also included to account for the inhibitory effects of methanol at the corresponding dilution levels.

### 2.3 Investigation of biofilm formation capacity

Biofilm formation was tested using crystal violet staining, which allows for the quantitative determination of total biomass as described by O’Toole [22] with some modifications. In the experiments assessing the effects of cyclodextrins, 50 μl of the sample was pipetted into the wells. For experiments using both farnesol and cyclodextrin, 25-25 µl of samples were measured into the wells, and then 150 μl of fungal suspension was added to the wells of a sterile, flat-bottomed polystyrene microtiter plate and incubated at 37 °C for 24 h. After incubation, the medium was decanted, and non-adherent cells were removed by washing the biofilm twice using a water-filled bath. 250 µl of methanol was added to the wells to fix the biofilm. After incubating for 15 min, the methanol was removed, and the plates were allowed to dry under laminar airflow. The wells were then filled with 250 μl of 0.1 % aqueous crystal violet solution and incubated for an additional 15 min in order to stain the cells. Excess stain was removed, and the washing steps were repeated carefully to avoid damaging the biofilm.

To solubilize the biofilm-bound crystal violet, 250 μl of 30 % acetic acid solution was added to the wells. After a 15-minute exposure, 250 μl of the extract was transferred to a new 96-well plate. Absorbance was measured at 544 nm using a Fluostar Optima microplate reader (BMG Labtech, Germany).

Based on the results, we calculated the relative biofilm formation compared to the control group that was not treated. The control value of '1′ serves as the basis for calculating relative changes in biofilm formation (see Fig.s).

### 2.4 Measurement of growth

In order to evaluate the microbial population's growth and ascertain whether the CDs exerted any cytotoxic effects, the optical density of the wells was measured. The DIALAB ELx800 ELISA Microplate Reader (Dialab GmbH, Wiener Neudorf, Austria) was used to ascertain the optical density at a wavelength of 630 nm, immediately following the plate assembly and after the incubation periods.

### 2.5 Microscopic investigation of morphological alterations induced by CDs

After samples were incubated for 24 h and stained with crystal violet, morphological investigations based on microscopic inspection were conducted to examine the morphologies of the treated *Candida albicans* cells. A Nikon Eclipse SI microscope with a TrueChrome 4K Pro camera and Mosaic™ V2.4 imaging software was used to visualize morphological changes under 400 × magnification (Auro-Science Consulting Kft., Budapest, Hungary) to determine whether cyclodextrins could impact the yeast-to-hyphae transition, representative images were taken.

### 2.6 Statistical analysis

Data were analyzed by analysis of variance (ANOVA) using StatSoft® Statistica 13.1 (TIBCO Software, Inc., Palo Alto, CA, USA) to determine whether the treatment effect was significant. One-way ANOVA was used to assess significant differences in growth and biofilm formation. Cochran's C-test was used to test the assumption of homogeneity of variance. Statistical analyses were performed at a significance level of p < 0.05. Fisher's least significant difference (LSD) test was used to compare the effects of different treatments. Significant deviations from the control are indicated by asterisks at the top of the columns.

## 3 Results

### 3.1 Effect of Cyclodextrins on Biofilm Formation of *Candida albicans*

Yeast cells were treated with nine different cyclodextrins at 37°C for 24 h to examine whether varying concentrations (ranging from 12.5 to 2.5 mM for eight cyclodextrins and 2.5 to 0.1 mM for native beta-cyclodextrin, due to its limited solubility) could affect biofilm formation. The results from crystal violet staining revealed that cyclodextrins have diverse effects on this process, exhibiting both stimulatory and inhibitory properties regarding biofilm formation. To distinguish between the effects of biofilm inhibition and those related to growth suppression, we assessed cell proliferation using an optical density-based method.

Measurements of optical density indicated a minimal impact across the tested cyclodextrins (detailed information can be found in Supplementary Table 1).

**Fig. 1:**
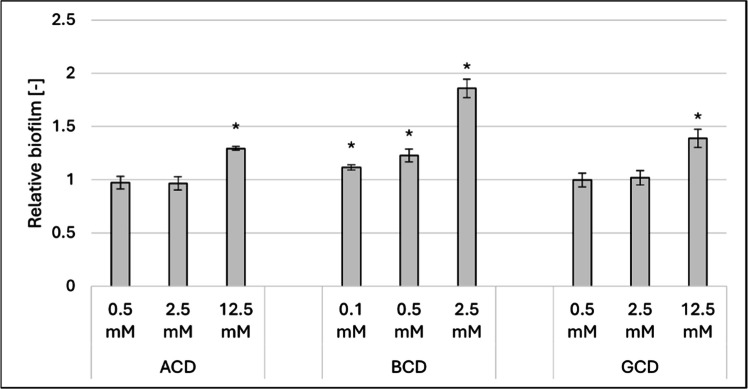
Effect of increasing concentrations of ACD, BCD and GCD on biofilm formation at 37°C. Significant effect compared to control is marked by an asterisk (*) (p < 0.05). Data represent averages of five replicates. The control value of “1” (without treatment) indicates a reference level of biofilm formation.

The effects of the three native cyclodextrins on biofilm formation are presented in Fig. 1.

For α-cyclodextrin and γ-cyclodextrin, a significant stimulation was observed at a concentration of 12.5 mM (29 % and 39 %, respectively). In contrast, β-cyclodextrin exhibited significant stimulation at much lower concentrations, with 0.1 mM already producing a noticeable effect. This stimulatory effect reached over 85 % at the highest tested concentration (2.5 mM), which concentration did not elicit a similar response in the other two native CDs.

**Fig. 2:**
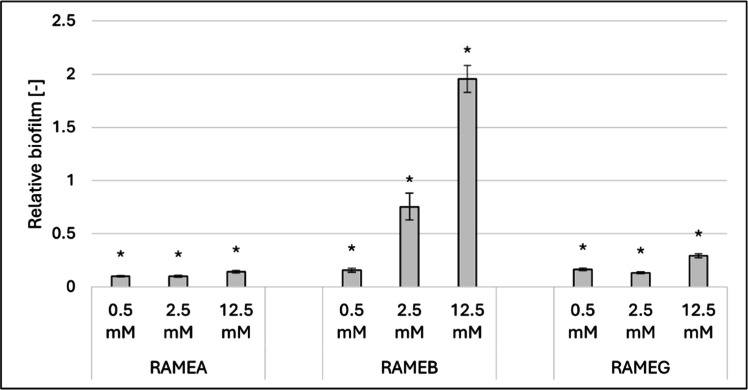
Effect of increasing concentrations of RAMEA, RAMEB and RAMEG on biofilm formation at 37°C. Significant effect compared to control is marked by an asterisk (*) (p < 0.05). Data represent averages of five replicates. The control value of “1” (without treatment) indicates a reference level of biofilm formation.

Among the randomly methylated derivatives (Fig. 2), the α- and γ-derivatives exhibited strong inhibitory effects on biofilm formation, with inhibition levels ranging from 71 % to 90 %. However, optical density measurements revealed minimal impact on growth, with none of the treatments causing more than a 20 % inhibition (Supplementary Table 1).

RAMEB demonstrated distinct behavior compared to the other two methylated derivatives. At lower concentrations, it inhibited biofilm formation (84 %–25 %), but as the concentration increased, this inhibition shifted to stimulation, reaching as high as 95 % at 12.5 mM.

**Fig. 3:**
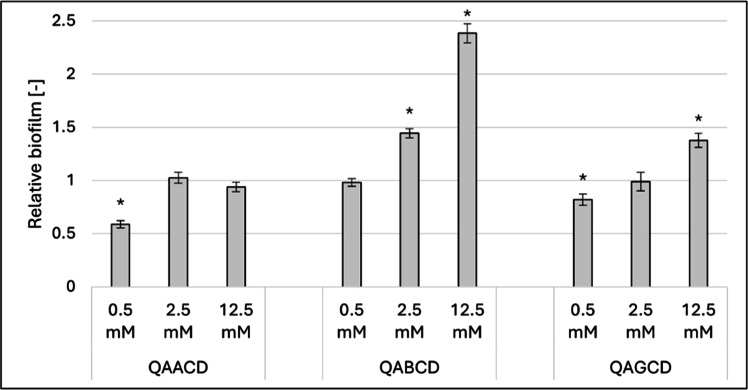
Effect of increasing concentrations of QAACD, QABCD and QAGCD on biofilm formation at 37°C. Significant effect compared to control is marked by an asterisk (*) (p < 0.05). Data represent averages of five replicates. The control value of “1” (without treatment) indicates a reference level of biofilm formation.

For the trimethyl-aminopropyl derivatives (Fig. 3), the behavior of the β-derivative, QABCD, stood out from its counterparts. QAACD at 0.5 mM inhibited biofilm formation as determined by crystal violet staining, but no significant effects were observed at higher concentrations.

QAGCD, when applied at 0.5 mM, caused an 18 % inhibition, whereas the highest concentration (12.5 mM) stimulated biofilm formation by 37 %. In contrast, QABCD did not cause any inhibitory effects but exhibited a remarkable stimulatory effect at the highest concentration, exceeding 138 %.

### 3.2 Effect of combined addition of cyclodextrins and farnesol on *Candida albicans* biofilm formation

Farnesol, a quorum-sensing molecule with potential as an antifungal agent, was added to *Candida albicans 10231* at three concentrations, both individually and in combination with various cyclodextrins. Following a 24-hour incubation period, its effects on growth and biofilm formation were assessed. Farnesol demonstrated concentration-dependent inhibitory effects on both growth and biofilm formation. At a concentration of 1250 µM, it exhibited an average cytotoxicity of 47 % and inhibited biofilm formation by 77 %. The impact of farnesol and its combinations with CDs on fungal growth is depicted in Supplementary Fig. 1.

**Fig. 4:**
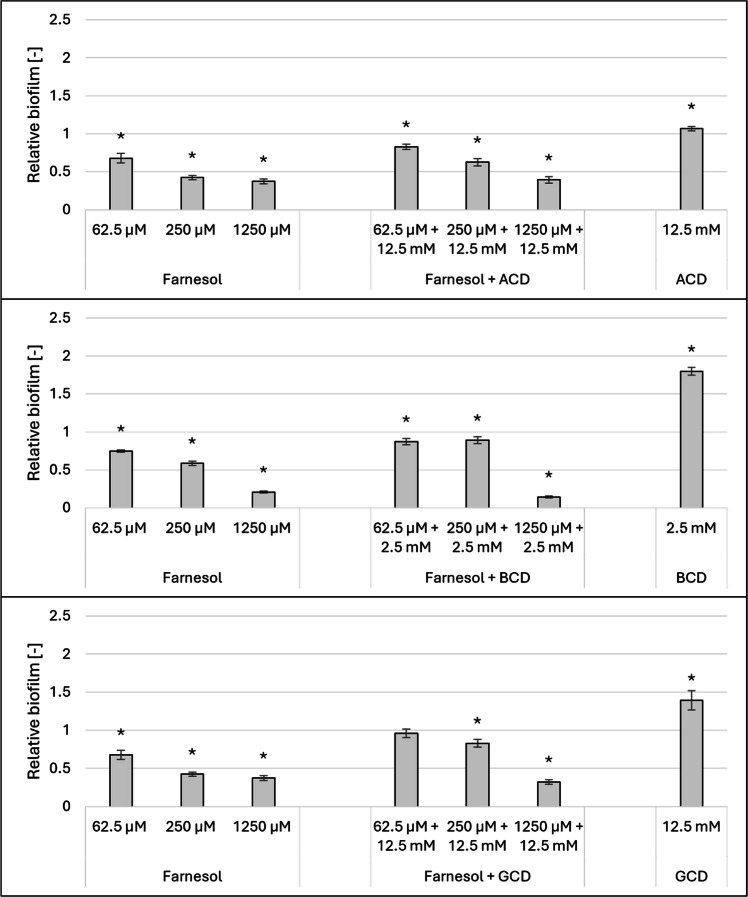
Effect of 12.5 mM ACD, BCD and GCD combined with increasing concentrations of farnesol on the biofilm formation of *Candida albicans (*at 37°C). Significant effect compared to control is marked by an asterisk (*) (p < 0.05). Data represent averages of five replicates. The control value of “1” (without treatment) indicates a reference level of biofilm formation.

The effects of the native CDs combined with farnesol on the biofilm formation are illustrated in Fig. 4.

Cyclodextrins co-added with farnesol were found to reduce the biofilm-inhibitory effect of farnesol at certain concentrations. Significantly more biofilm was formed when ACD was added to the system alongside 250 µM or 62.5 µM farnesol, resulting in a 22–47 % increase in biofilm formation. A similar trend was observed with BCD and GCD: when these cyclodextrins were combined with 250 µM or 62.5 µM farnesol, biofilm formation increased by 17–51 % and 41–95 %, respectively, compared to treatments with farnesol alone.

In contrast, the addition of native cyclodextrins did not reduce the biofilm-inhibitory effect of farnesol at a higher concentration of 1250 µM.

**Fig. 5:**
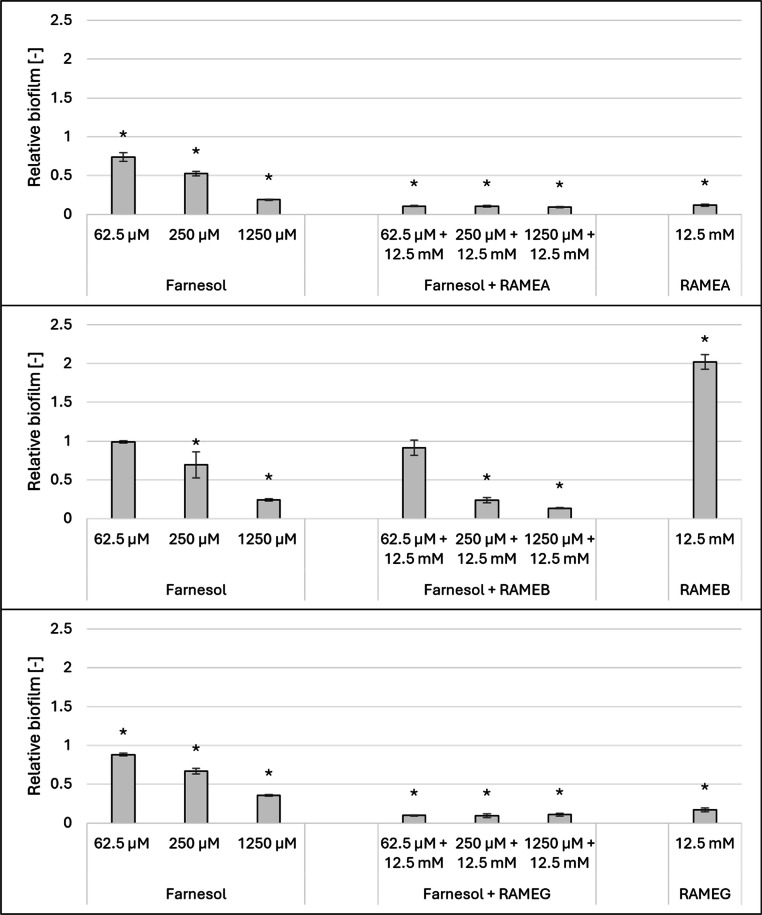
Effect of 12.5 mM RAMEA, RAMEB and RAMEG combined with increasing concentrations of farnesol on the biofilm formation of *Candida albicans (*at 37°C). Significant effect compared to control is marked by an asterisk (*) (p < 0.05). Data represent averages of five replicates. The control value of “1” (without treatment) indicates a reference level of biofilm formation.

In Fig. 5. we illustrated the effects of RAMEA, RAMEB, and RAMEG on biofilm formation in *C. albicans.*

In all experiments, increasing concentrations of farnesol resulted in a concentration-dependent inhibition of biofilm formation. RAMEA, when applied alone, exhibited a pronounced inhibitory effect on biofilm formation, reducing it by 88 %. This effect was consistently maintained when RAMEA was used in combination with farnesol. A similar trend was observed with RAMEG, which not only maintained its inhibitory effect in the combined treatment but significantly enhanced it compared to either CD or farnesol alone, suggesting a potential synergistic effect. By contrast, 12.5 mM RAMEB alone demonstrated a notable stimulatory effect, enhancing biofilm formation by 102 %.

However, this stimulatory effect was not evident when RAMEB was combined with farnesol. Moreover, treatments containing both farnesol and 12.5 mM RAMEB resulted in lower biofilm formation compared to treatments with farnesol alone at both 250 µM and 1250 µM concentrations.

**Fig. 6:**
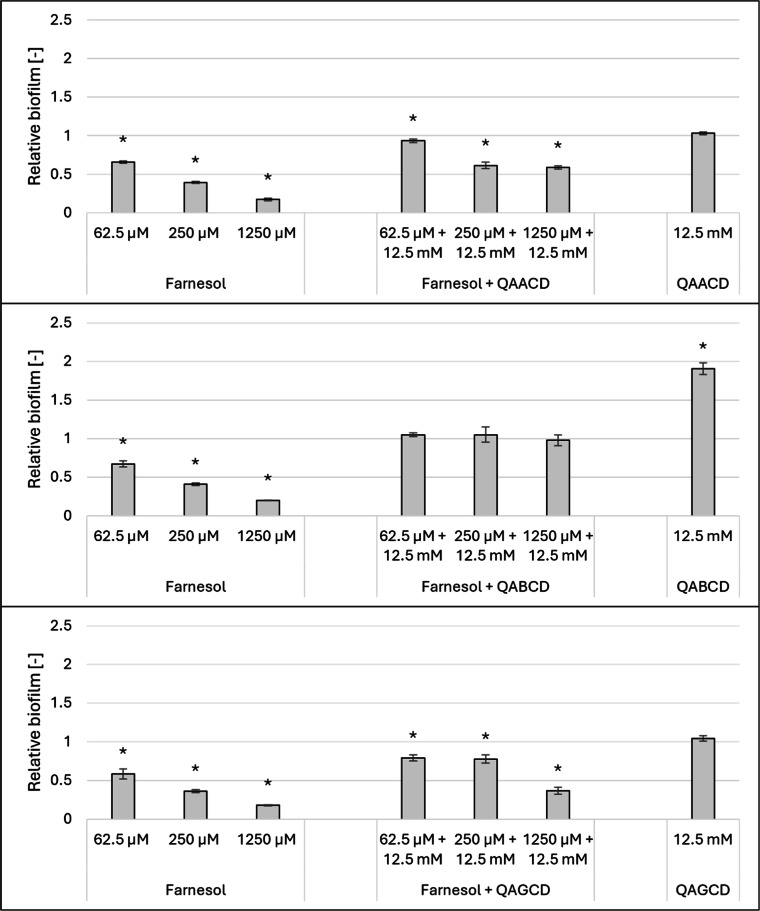
Effect of 12.5 mM QAACD, QABCD and QAGCD combined with increasing concentrations of farnesol on the biofilm formation of *Candida albicans (*at 37°C). Significant effect compared to control is marked by an asterisk (*) (p < 0.05). Data represent averages of five replicates. The control value of “1” (without treatment) indicates a reference level of biofilm formation.

For the trimethyl-aminopropyl derivatives (Fig. 6), QAACD and QAGCD alone did not influence biofilm formation at a concentration of 12.5 mM. However, in combination treatments, these derivatives significantly attenuated the inhibitory effect of farnesol.

In the case of 12.5 mM QABCD, the significant biofilm-stimulating effect of the cyclodextrin was evident, and similar to the other derivatives, it also reduced the inhibitory impact of farnesol in the combined treatments.

### 3.3 Effect of cyclodextrins on the morphology of *Candida albicans*

The impact of cyclodextrins on the morphology of *Candida albicans* was examined. Following a 24-hour incubation at 37 °C with various cyclodextrins, the biofilm-adhered cells were stained with crystal violet (CV) and analyzed under a microscope at 400x magnification to assess cell shape and morphological differences compared to the control.

**Fig. 7:**
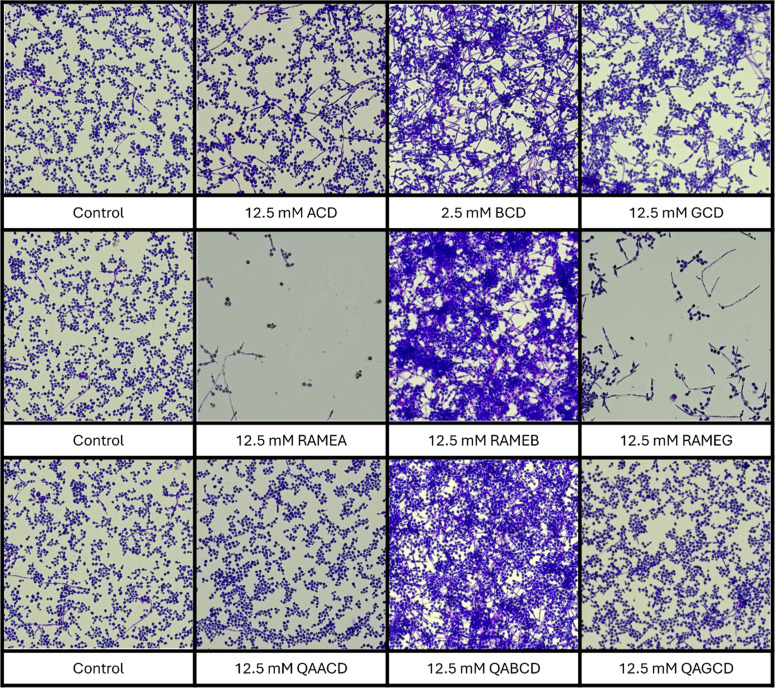
Morphological changes observed at 400x magnification.

As shown in Fig. 7, similar to their biofilm-stimulatory effects, BCD, RAMEB, and QABCD exhibited significant morphological differences compared to the control cells. Additionally, for RAMEA and RAMEG, which demonstrated strong biofilm-inhibitory effects, fewer cells were observed under the microscope, consistent with their impact on biofilm formation.

For QAACD and QAGCD, no morphological differences were observed, which is consistent with their minimal influence on biofilm formation. Overall, except for QAACD and QAGCD, an increase in the number of hyphal forms was noted for the cyclodextrins in comparison to the control.

RAMEB exhibited unique behavior, producing dense biofilms and numerous hyphal forms at a concentration of 12.5 mM. However, at lower concentrations of 2.5 mM and 0.5 mM, the quantity of biofilm was significantly reduced, while the large number of hyphal forms remained unchanged (Supplementary Fig. 2).

## 4 Discussion

In this study, we investigated the effects of cyclodextrins on *Candida albicans* quorum-sensing regulated processes, specifically focusing on biofilm formation and morphological changes. One of our key objectives was to assess the possibility of free CDs being able to reduce the viability and biofilm formation capacity of pathogenic yeast (*C. albicans*) without other active ingredients.

The results highlight the diverse impact of cyclodextrins on *Candida albicans* biofilm formation, revealing both stimulatory and inhibitory effects that vary with the concentration and structure of the CDs. Among the three native CDs, ACD and GCD significantly stimulated biofilm formation at 12.5 mM, while BCD exhibited stimulation at much lower concentrations, with the effect peaking at over 85 % at 2.5 mM. For the randomly methylated derivatives, RAMEA and RAMEG strongly inhibited biofilm formation (71 %–90 %), with minimal effects on cell growth. Interestingly, RAMEB displayed a concentration-dependent dual behavior, shifting from inhibition at lower concentrations to stimulation at higher concentrations (up to 95 % at 12.5 mM). The trimethyl-aminopropyl derivatives also demonstrated unique patterns. While QAACD and QAGCD showed mild inhibitory or stimulatory effects depending on the concentration, QABCD exhibited a pronounced stimulatory effect at 12.5 mM, exceeding 138 %. These findings suggest that the CDs composed of seven glucose units (BCD, RAMEB, and QABCD) consistently showed greater stimulatory effects on biofilm formation compared to their six- and eight-unit counterparts.

BCD has the poorest solubility of the 9 CDs, because of its rigid structure, and its intramolecular hydrogen bonds limit its ability to interact with water molecules. The solubility of CDs can be significantly increased when the hydroxyl groups on the CD molecule's outer surface are randomly substituted [38], that is the reason why we also used RAMEB and QABCD as potential molecules to influence the QS mediated processes in *C. albicans.*

We also investigated the impact of cyclodextrins on the morphology of *Candida albicans*. The cells can transition from a yeast state to pseudohyphal and hyphal forms, a transformation regulated by factors such as temperature, pH, carbon dioxide concentration, nutrient composition, and cell population density [37]. We found that in addition to influencing biofilm formation, the CDs also induced changes in cell morphology, a phenomenon that appeared to depend on multiple factors, including the size of the internal cavity, the presence of substitution groups, and the concentration of the CDs.

It has been demonstrated that cyclodextrins alone are capable of influencing quorum sensing regulated biofilm formation in *Candida albicans* at 37 °C. Nevertheless, the mechanisms by which these effects occur have not yet been fully elucidated.

Ramage et al. [25] were the first to demonstrate that biofilm formation, as measured by XTT in *Candida albicans* collection strains 3153A and SC5314, was inhibited in a concentration-dependent manner by 300 µM–3 mM farnesol when it was added at the very beginning of the incubation process. Farnesol inhibits hyphal formation, thereby preventing the transition of the yeast form into the hyphal morphology [10]. Suppressing the yeast-to-hyphal transition interferes with a key virulence factor, as hyphal forms play a central role in tissue invasion and biofilm architecture. Farnesol also induces apoptosis in *C. albicans* [32].

Although the *Candida albicans* ATCC 10231 yeast produces farnesoic acid, an autoregulatory substance capable of regulating morphological transition [19,21,26], this strain is also used in research relating to the biofilm formation in the presence of farnesol [8,31]. In accordance with the results of our present study, Fernandes et al. [8] and Sebaa et al. [31] have also demonstrated the inhibitory effect of farnesol on the biofilm formation of this particular yeast strain. However, the effect of cyclodextrin, either on its own or in combination with farnesol, on the QS-regulated processes of this yeast has not yet been studied.

Our results with random methylated derivatives suggest that these CDs possess inherent bioactive properties that significantly influence fungal physiology.

The results may also be explained by cyclodextrin's ability to complex other signal molecules involved in quorum sensing, such as tyrosol [2]. Tyrosol, particularly during the early stages of biofilm development, stimulates hyphal formation and biofilm growth, consequently, together with farnesol, tyrosol is a molecule that plays a key role in quorum sensing regulated processes in *Candida* species [31]. Cyclodextrins may be able to improve and reduce the availability of tyrosol by complexing it, but the mechanisms of this process are not yet known and need to be explored in future studies.

Furthermore, a combination of CDs and farnesol, a known antifungal molecule, was investigated to ascertain whether cyclodextrins could influence the effect of farnesol on the biofilm-forming ability of *C. albicans* for example by affecting the solubility and bioavailability of this antifungal agent. Our hypothesis is based on the examples in the literature of farnesol being complexed by cyclodextrins [6,29,33,34].

At the concentrations tested in our study, RAMEA and RAMEG exerted such strong inhibitory effects that the addition of farnesol did not result in any further changes in inhibition. To explore this phenomenon further, we plan to examine the effects of these CDs and farnesol at lower concentrations to determine whether their combined application with farnesol might synergistically enhance its biofilm-inhibitory effects, as RAMEB and RAMEG appeared to enhance the inhibitory effect of farnesol on biofilm formation.

As demonstrated in present literature, farnesol has been shown to have a number of health benefits, including antitumor and antimicrobial properties [5]. Such synergism could prove advantageous for developing technologies against *Candida albicans.*

The findings of the present study also demonstrate that the effects of a randomly methylated derivative of gamma-cyclodextrin (RAMEG) are distinctive and highly significant. Compared to native ACD and BCD, GCD possesses the largest cavity with hydrophobic character, the highest water solubility and most beneficial toxicological characteristic [30]. In our study, RAMEG showed high bioactive potential, manifested by its extraordinary and significant biofilm formation inhibitory activity both alone and in combination with farnesol.

The high inhibitory effect of gamma cyclodextrin may be related to their ability to destabilize the membrane, as Ruiz et al. [27] observed that GCD exhibited a synergistic effect in membrane destabilization when combined with amphotericin B, although in our study only the random methylated derivative showed significant inhibitory effect, and mainly on biofilm formation.

In addition to the promising results, the parameters of both the bioactivity of CD and the mechanism of action and application of CD-based farnesol carriers to reduce biofilm formation require further investigation. A deeper understanding of these processes could serve as a valuable tool for influencing the quorum sensing-regulated processes especially biofilm formation with Candida species. The increase in the occurrence of fungal infections on a global scale, coupled with the rise in the occurrence of antifungal resistance, underscores the necessity for novel research to identify antifungal molecules.

## 5 Conclusion

The present study revealed the bioactive potential of specific cyclodextrins, observing both stimulatory and inhibitory effects of cyclodextrins on *C. albicans* biofilm formation, depending on the structure and concentration of the cyclodextrins.

The study demonstrated that randomly methylated α-CD (RAMEA) and randomly methylated γ-CD (RAMEG) exhibited significant antifungal properties, evidenced by a reduction in the biofilm formation capacity of *C. albicans*. These findings suggest that these cyclodextrins could serve as a promising alternative for the treatment of candidiasis. Furthermore, RAMEB and RAMEG was found to enhance the antifungal activity of farnesol against *C. albicans*. Consequently, its synergistic effect with farnesol may offer an excellent opportunity to produce a reduced dose but more effective formulation.

In addition to the encouraging results, further investigation is required into the bioactivity of CD and the mechanism of action and application of CD-based farnesol carriers to reduce biofilm formation. The testing of additional concentrations and combinations may facilitate the development of effective antifungal bioactive molecules and complexes.

## CRediT authorship contribution statement

**Rita Márton:** Writing – review & editing, Writing – original draft, Visualization, Methodology, Investigation, Funding acquisition, Formal analysis, Data curation, Conceptualization. **Hanna Hermann:** Methodology, Investigation. **Virág Tünde Kiss:** Methodology, Investigation. **Éva Fenyvesi:** Validation, Supervision, Conceptualization. **Lajos Szente:** Validation, Supervision, Conceptualization. **Mónika Molnár:** Writing – review & editing, Writing – original draft, Supervision, Resources, Project administration, Funding acquisition, Formal analysis, Conceptualization.

## Declaration of competing interest

The authors declare the following financial interests/personal relationships which may be considered as potential competing interests:

Rita Márton reports financial support was provided by Richter Gedeon Talentum Foundation. Mónika Molnár reports financial support was provided by Ministry of Culture and Innovation of Hungary from the Hungarian National Research, Development and Innovation Fund. If there are other authors, they declare that they have no known competing financial interests or personal relationships that could have appeared to influence the work reported in this paper.

## Data Availability

Data will be made available on request.

## Appendix Supplementary materials

Image, application 1
