# Supplementary material for: Cyclodextrins in action: Modulating candida albicans biofilm formation and morphology
Source: Biotechnol Rep (Amst). 2025 Aug 9;47:e00912. doi: 10.1016/j.btre.2025.e00912 (PMC12398212; doi:10.1016/j.btre.2025.e00912)
Supplement: Supplementary file 1 [file mmc1.docx]

**Supplementary Materials**

**for**

**Cyclodextrins in Action: Modulating *Candida albicans* Biofilm Formation and Morphology**

Rita Márton^1^, Hanna Hermann^1^, Virág Tünde Kiss^1^, Éva Fenyvesi^2^, Lajos Szente^2^ and Mónika Molnár^1,*^

^1^Department of Applied Biotechnology and Food Science, Budapest University of Technology and Economics, 1111 Budapest, Hungary

^2^CycloLab Cyclodextrin R & D Laboratory Ltd., 1097 Budapest, Hungary

*Correspondence: [molnar.monika@vbk.bme.hu](mailto:molnar.monika@vbk.bme.hu)

**Supplementary Table 1.** The impact of cyclodextrins on the growth of *Candida albicans*. The growth observed in the control group is designated as 1 for comparative purposes. Significant effect compared to control is marked by asterisk (*) (p < 0.05). Data represent averages of five replicates.

| Cyclodextrin effect on the growth of *Candida albicans* | | | |
| --- | --- | --- | --- |
|  | Cyclodextrin concentration | | |
|  | 0.5 mM | 2.5 mM | 12.5 mM |
| ACD | 1.04 (±0.03) | 1.00 (±0.06) | 0.98 (±0.03) |
| BCD | 1.01 (±0.03) | 1.01 (±0.03) | 1.00 (±0.04) |
| GCD | 1.02 (±0.03) | 1.00 (±0.06) | 1.08 (±0.04) * |
| RAMEA | 1.17 (±0.07) * | 1.16 (±0.04) * | 1.15 (±0.04) * |
| RAMEB | 1.18 (±0.06) * | 1.15 (±0.06) * | 1.19 (±0.04) * |
| RAMEG | 1.10 (±0.04) * | 1.20 (±0.02) * | 1.20 (±0.04) * |
| QAACD | 1.07 (±0.06) | 1.10 (±0.04) * | 1.08 (±0.06) |
| QABCD | 1.07 (±0.05) | 1.11 (±0.05) * | 1.20 (±0.05) * |
| QAGCD | 0.99 (±0.02) | 1.06 (±0.05) | 1.10 (±0.04) * |

**
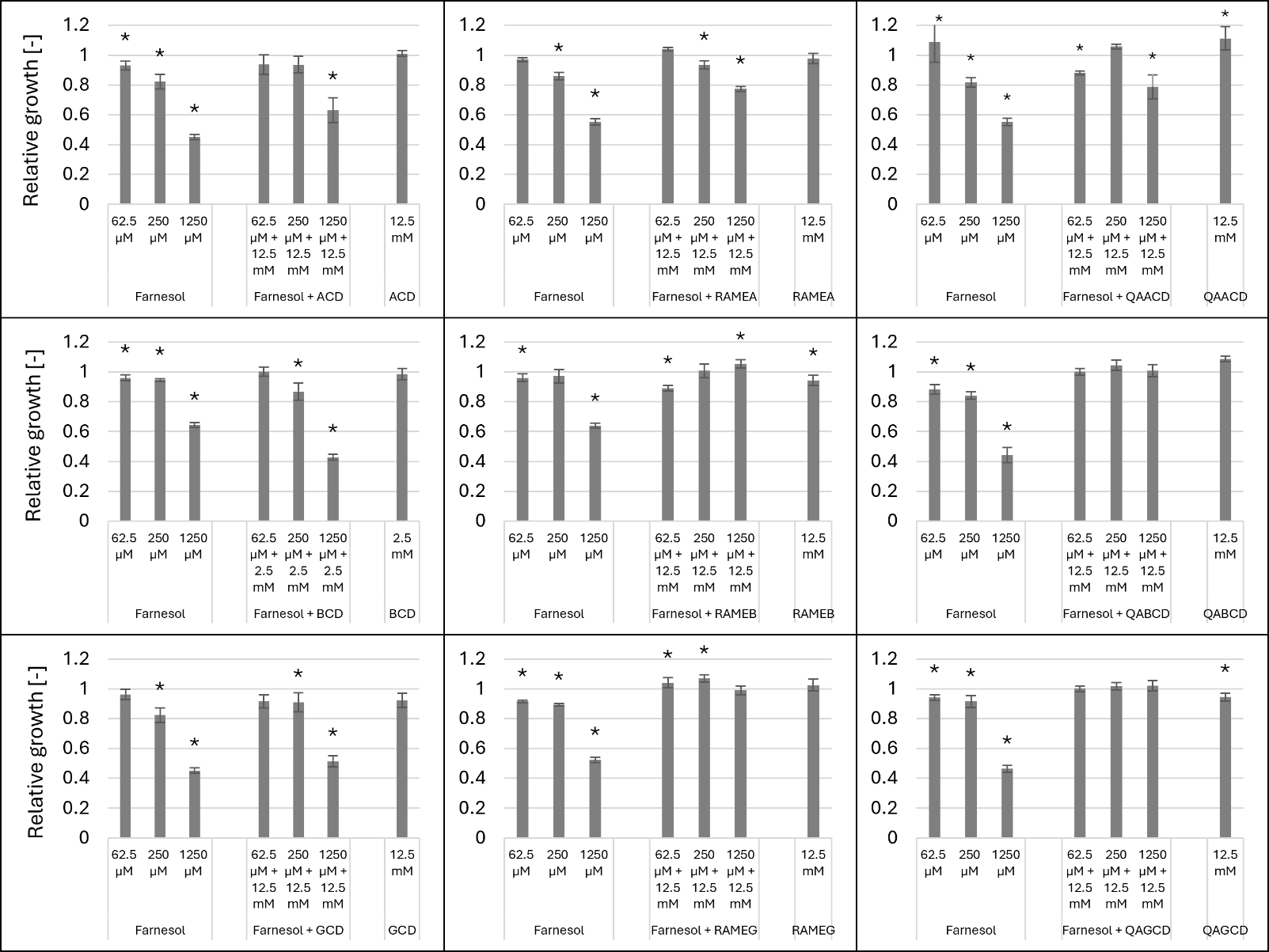
**

**Supplementary Figure 1.** Effect of increasing concentrations of farnesol plus ACD, BCD, GCD, RAMEA, RAMEB, RAMEG, QAACD, QABCD and QAGCD on the relative growth of *Candida albicans* at 37 ^o^C. Significant effect compared to control is marked by an asterisk (*) (p < 0.05). Data represent averages of five replicates.


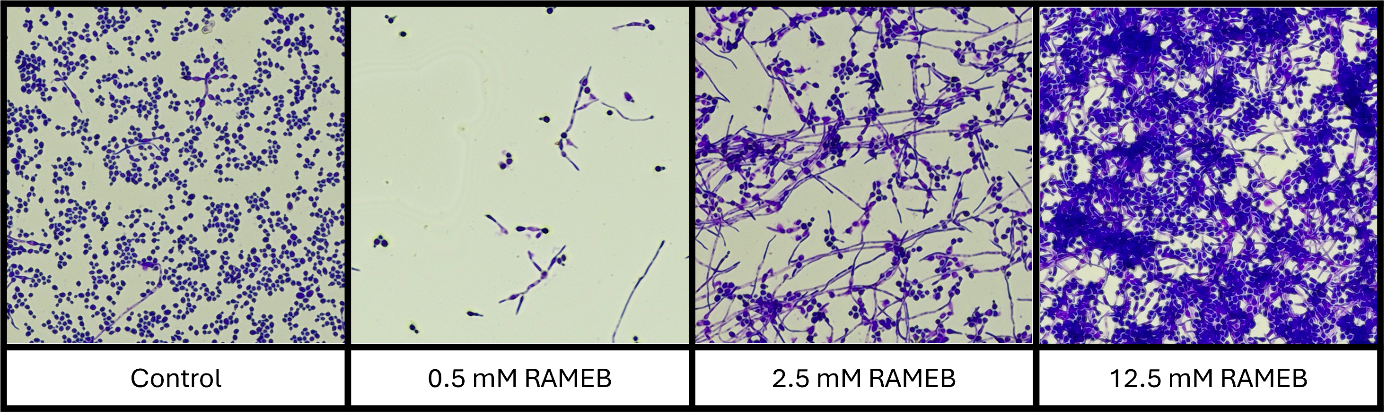


**Supplementary Figure 2.** Effect of increasing concentrations of RAMEB on the morphology of *Candida albicans* (the images were taken at 400x magnification level).
